# Puerarin’s multidimensional antidepressant action: decoding the gut-liver-brain axis through gut microbiota, hepatic homeostasis, and neuroimmune regulation

**DOI:** 10.3389/fnins.2026.1698851

**Published:** 2026-02-03

**Authors:** Lufen Ye, Linlu Peng, Jiaojiao Tian, Hao Ma

**Affiliations:** 1 School of Pharmacy, Yichun University, Yichun, Jiangxi, China; 2 School of Chemical and Biological Engineering, Yichun University, Yichun, Jiangxi, China; 3 School of Basic Medical Sciences, Yichun University, Yichun, Jiangxi, China

**Keywords:** chronic restraint stress (CRS), depression, intestinal flora, puerarin, TLR4

## Abstract

**Background:**

Puerarin is a flavonoid bioactive component extracted from the Chinese herb radix puerariae, which has been reported to have anti-inflammatory and neuroprotective effects and is a potential drug for the treatment of neuroinflammatory diseases. There is increasing evidence that the gut-liver-brain axis is closely related to neurological disorders. However, studies on the use of puerarin for the treatment of depression based on gut-liver-brain axis-mediated inflammatory injury have not been reported.

**Methods:**

In the present study, a 4-week chronic restraint stress (CRS) mouse depression model was established. Place the mice in 50 mL centrifuge tubes for restraint. The tubes should be perforated with 15–20 small holes to ensure adequate ventilation. The restraint period is from 9:00 a.m. to 1:00 p.m. daily, during which food and water are withheld. Based on the results of previous studies, the better antidepressant dose of puerarin, 100 mg/kg, was chosen, and fluoxetine was used as a positive control to investigate the intervention effect and potential mechanism of puerarin on depression. All of the aforementioned drugs were administered via oral gavage. Sucrose preference test (SPT), tail suspension test (TST), open field test (OFT), novelty suspended feeding test (NSFT) and forced swimming test (FST) were used to observe the behavioral changes in mice to assess the antidepressant effects. The microbial composition of the intestinal tract was analyzed using 16S rRNA gene sequencing. Histopathological changes in colon and liver were also observed by HE staining method. The levels of lipopolysaccharide (LPS) in colon, serum, liver and prefrontal cortex (PFC) and the levels of 5-hydroxytryptamine (5-HT) in prefrontal cortex were detected by enzyme-linked immunosorbent assay (ELISA). The method was developed for the detection of 5-HT in the prefrontal cortex. The serum levels of glutamate transaminase (AST) and alkaline phosphatase (ALP) were measured by microplate assay. Finally, the expression of brain-derived neurotrophic factor (BDNF), TLR4, MYD88, p-IκB-α, and p-p65 proteins were determined by immunoblotting assay (Western Blot, WB) in mice with PFC.

**Results:**

Puerarin was effective in alleviating CRS-induced depression-like behaviors measured in SPT, TST, FST and NSFT in mice. Compared with the CRS model group, puerarin increased the rate of sugar-water preference in the SPT and shortened the cumulative immobility time in the TST and FST as well as the ingestion latency in the NSFT in depressed mice. In addition, puerarin administration ameliorated CRS-induced gut microbiota dysbiosis in mice, elevating the abundance of Lactobacillaceae, Lactobacillus spp. Decreased the relative abundance of Ruminococcaceae, Ruminococcus, Desulfovibrionaceae, and Prevotella spp. Puerarin also reduced LPS, AST and ALP levels, improved damaged colon and liver tissues, inhibited neuroinflammatory damage mediated by the TLR4/MYD88/NF-κB signaling pathway, and up-regulated the levels of 5-HT and BDNF in the prefrontal cortex of the mice, thereby reversing CRS-induced depressive-like behaviors in depressed mice.

**Conclusion:**

Puerarin can improve CRS-induced depression in mice by regulating the gut-liver-brain axis and its related molecules. For example, it can regulate CRS-induced intestinal flora disorders and intestinal permeability, thereby reducing systemic LPS levels and the relative levels of AST and ALP, inhibiting the activation of the TLR4/MYD88/NF-κB signaling pathway by LPS, thereby reducing neuroinflammatory damage, and ultimately improving the depressive symptoms of CRS mice.

## 1 Introduction

Depression is a neuropsychiatric disorder accompanied by clinical symptoms such as slow thinking, reduced interest, negative pessimism, and even self-harm and suicidal behavior in severe cases. At present, the emotional disorders caused by the loss of liver detachment are more similar to the onset and symptomatic manifestations of depression. Depression has seriously affected patients’ physical and mental health, living standards and occupational ability, bringing a heavy burden to patients, their families and even the whole society. According to the World Health Organization (WHO), more than 300 million people around the world are suffering from depression, and the number of patients with depression increased by 18.4% from 2005 to 2015, and the data of this type of common mental disorders show an increasing trend (World Health Organization, 2017). Depression is expected to be one of the largest contributors to the global burden of disease by 2030 (Whiteford et al., 2013). Therefore, the development of safe and effective antidepressants is urgent. However, the pathogenesis and pathophysiology of depression is more complex and has not yet been fully elucidated, and a series of studies have shown that it may involve a variety of influencing factors, such as neurobiochemical, social, genetic, and psychological, etc. In the absence of a complete understanding of the etiology of the disease, the research and development of antidepressant medications can not be sustained and targeted improvement of patients’ symptoms of depression, and there is no treatment that is currently considered to be curative for depression (Zhou et al., 2021). Therefore, new research strategies are needed to explore the mechanisms of depression pathology and the development of novel antidepressant drugs.

The pathogenesis of depression mainly involves the following aspects: (1) monoamine neurotransmitter hypothesis, this type of hypothesis is currently more widely accepted hypothesis of the etiology of depression, the theory that depression occurs mainly due to the level or dysfunction of monoamine neurotransmitter concentration in the synaptic gap of the central nervous system. For example, norepinephrine (NE) levels and 5-hydroxytryptamine (5-HT) levels are decreased in the brain of depressed patients. Monoamine oxidase inhibitors or 5-HT reuptake inhibitors exert antidepressant effects by inhibiting the degradation of monoamines in the brain or increasing the concentration of monoamine neurotransmitters in the synaptic gap in the brain (Yohn et al., 2017). Monoamine neurotransmitters such as 5-HT, NE, and dopamine (DA) are involved in a number of physiological responses related to the nervous system, such as emotional responses, mental activity, and thermoregulation. (2) Hypothalamic-pituitary-adrenal (HPA) axis dysfunction hypothesis, HPA is closely related to the pathophysiology of a wide range of mood and cognitive disorders in the body, and abnormalities in its regulation usually affect the onset and progression of depression, and patients with depression often show dysfunction of the HPA axis (Woelfer et al., 2019; Murri et al., 2014). It has even been pointed out that the cognitive ability of depressed patients is negatively correlated with higher cortisol (Keller et al., 2017). (3) Neurotrophic hypothesis, brain-derived neurotrophic factor (BDNF) is a kind of neurotrophic factor. BDNF has a key role in cell differentiation, neuronal development and survival, synaptogenesis and synaptic plasticity (Greenberg et al., 2009). Studies have shown that abnormal BDNF levels are often present in the hippocampal tissue of chronically stressed mice and depressed patients (Castrén and Rantamäki, 2010). (4) Immuno-inflammatory hypothesis, studies have shown that peripheral blood and central pro-inflammatory cytokines are increased in patients with depression, and peripheral inflammatory factors can pass the blood-brain barrier (BBB) and participate in neuroinflammatory activities in the centre, thus participating in the development of depression-like symptoms in patients (Benedetti et al., 2020). (5) Microbiota-gut-brain (MGB) axis hypothesis, in recent years, more and more evidence supports the involvement of intestinal flora in the development of organic diseases, and reveals the relationship between intestinal flora and many neuropsychiatric disorders, which mainly believes that depression is closely related to the imbalance of intestinal flora, and that depressive behaviors are usually accompanied by changes in the intestinal microbial diversity and altered relative abundance (Schaub et al., 2022).

In recent years, there has been increasing evidence of gut microbiological alterations in depressed patients, but the relationship between depression and gut dysbiosis has not yet been fully elucidated. A large amount of gut flora exists in the animal gut and is essential for human gastrointestinal health. It is also important for the growth, development and function of the central nervous system, and therefore the gut flora is often referred to as the “second brain,” which influences the growth, development and function of the brain under both pathological and physiological conditions (Sochocka et al., 2019). In recent years, a growing number of studies have shown differences in the composition of the gut microbiota between healthy and depressed individuals. The results of fecal flora analysis in depressed patients and healthy controls showed increased levels of, for example, Bacteroidetes and Proteobacteria in the feces of depressed patients. While the levels of Firmicutes were decreased (Jiang et al., 2015). Increased abundance of Bacteroidetes and decreased abundance of Firmicutes have also been observed in animal models of depression (Yu et al., 2017). Germ-free (GF) mice that received fecal microbiota transplants (FMT) from depressed patients were more likely to develop depressive-like behaviors than GF mice that received transplants of “healthy microbiota” from healthy controls. Mice with “depressed microbiota” mainly showed disturbances in microbial genes involved in carbohydrate and amino acid metabolism and host metabolites (Zheng et al., 2016). Another study found that when the microbiota of depressed mice was transplanted into mice depleted of gut flora by antibiotic administration, the recipient mice exhibited depressive behaviors and reduced adult hippocampal neurogenesis (Chevalier et al., 2020). Furthermore, it was demonstrated that by administering the probiotic *Lactobacillus kefiranofaciens* ZW3 to chronic stress-induced depressed mice. ZW3 modulates stress-induced biochemical disturbances of the HPA axis, the immune system and tryptophan metabolism, as well as regulates the composition of the intestinal microbiota, and is effective in ameliorating chronic stress-induced depressive-like behavior induced by chronic stress (Sun et al., 2019). These findings contribute to a better understanding of the changes in the composition of the fecal microbiota in depressed patients, suggesting a predominance of certain potentially harmful flora or a decrease in beneficial bacteria.

Some studies have found interactions between the gut microbiota, inflammation and the brain, and have proposed the concept of the microbiota-gut-inflammatory vesicle-brain axis. The gut microbiota and its metabolites are involved in the regulation of neuroinflammation affecting brain function by stimulating the activation of inflammatory vesicles, contributing to the maturation of caspase-1 and the activation of interleukin-1β (IL-1β) (Wong et al., 2016). Disturbances in intestinal flora not only lead to alterations in intestinal barrier function and intestinal permeability, affecting gastrointestinal epithelial cells and the immune system, but also play an important role in the regulation of body behaviors and brain functions, including stress response, emotional behavior, pain modulation, and ingestive behaviors (Yang et al., 2018). Altered intestinal permeability leads to lipopolysaccharide (LPS) and bacterial translocation to the portal (and via shunting to the systemic) circulation, resulting in immune activation and eventual liver injury as well as systemic inflammation (Shi et al., 2020). LPS is the main component that forms the outer wall of Gram-negative bacteria and consists of three regions: the O-antigen, the core polysaccharide, and lipid A. LPS forms a protective barrier around the bacteria to evade the action of antibiotics (Kim et al., 2016). When the Gram-negative bacterial population is increased, the highly pro-inflammatory LPS can be released, LPS can be transported across the damaged intestinal barrier through the portal vein or lymphatics to the liver, and when liver damage results in partial loss of detoxification, some of the un-removed LPS enters the corpuscular circulation, and at low concentrations, LPS does not cross the BBB but it stimulates the production of pro-inflammatory cytokines, inhibits neurogenesis and reduces brain volume, whereas high concentrations of LPS lead to partial BBB destruction so that the toxin can subsequently reach areas of the brain such as the cortex and thalamus (Boeri et al., 2019). LPS acts as an agonist of toll-like receptor 4 (TLR4) and acts on the TLR4 receptor, activating the TLR4/MYD88/NF-κB signaling pathway and increasing the expression of pro-inflammatory cytokines such as interleukin-6 (IL-6), IL-1β and tumour necrosis factor-α (TNF-α) and other pro-inflammatory cytokines expression, which leads to low levels of chronic inflammation in the body (Guo et al., 2021). Vagal output from the liver has been found to be associated with changes in gut microbial composition, neuroinflammation and plasticity (Zhang et al., 2021). Liver injury is strongly associated with the severity of gut microecological dysregulation (Bajaj et al., 2014). It has also been shown that cirrhosis and hepatic encephalopathy are associated with alterations in the “gut-liver-brain axis.” Transplantation of fecal flora from patients with cirrhosis resulted in higher neuroinflammation in germ-free mice, suggesting that the gut microbiota mediates neuroinflammation, and that changes in liver function and alterations in the composition of gut microbes lead to brain dysfunction (Liu et al., 2020). Therefore, the role of the liver in the gut-brain axis should not be overlooked while focusing on the important role of the gut-brain axis in disease development.

Currently, clinical treatments for depression include antidepressant pharmacotherapy, psychological interventions, and physical therapies such as electroconvulsive therapy (ECT). Psychological interventions are often used as the main treatment for mild patients, but pharmacotherapy is often the main medical option for moderately to severely depressed patients. Common antidepressant drugs include selective serotonin reuptake inhibitors (SSRIs), represented by fluoxetine, sertraline, etc. And serotonin noradrenalin reuptake inhibitor (SNRI), represented by venlafaxine, duloxetine, etc. It is mainly used to increase the synaptic gap between the synapses of the brain and the brain. They mainly exert antidepressant effects by increasing the concentration of monoamine neurotransmitters such as 5-HT and NE in the synaptic gap (Hillhouse and Porter, 2015). However, most of the existing antidepressants are prone to drawbacks such as drug tolerance, slow onset of action, poor patient compliance and numerous side effects (e.g., nausea, insomnia, etc.) (Khawam et al., 2006). Although some animal studies have shown that fluoxetine can regulate the levels of BDNF and 5-HT in the hippocampus and enhance neuronal plasticity, which in turn improves depression-like behavior in mice (Song, 2019). However, the application of fluoxetine in the treatment of depression still exists problems such as slow onset of action, large individual differences, and large side effects in some patients, which greatly limits its clinical application. Therefore, it is necessary to elucidate the pathogenesis of depression and find new antidepressants with fewer side effects, rapid onset of action and stable efficacy. Recent studies have found that a variety of traditional Chinese medicines can prevent and treat depression by regulating intestinal flora. For example, the TCM formula Xiaoyao powder attenuated chronic restraint stress (CRS)-induced depression-like behavior in rats by modulating the intestinal flora and its metabolite short-chain fatty acids (SCFAs), while Xiaoyao powder was shown to reduce the abundance of Desulfovibrio spp. and thus reduce the release of inflammatory factors (Zhu et al., 2019). Another study found that the extraction of total extract of the active parts of *Schisandra chinensis* and lignans significantly inhibited the TLR4/NF-κB/IKKα signal transduction pathway. It also up-regulated the concentrations of butyric and propionic acids in mouse blood and restored the LPS-induced reduction in the diversity of mouse gut microbiota (Yan et al., 2021). With the progress in the ongoing study of the relationship between intestinal flora and disease, it has been found that the regulation of intestinal microecological balance may be one of the important therapeutic strategies for the prevention and treatment of depression. It provides a new perspective to explore the antidepressant mechanism of Pueraria.

Puerarin, also known as *Pueraria lobata*, is a flavonoid bioactive component extracted from the Chinese herb *Pueraria lobata*. Numerous studies have shown that puerarin has an extremely wide range of pharmacological effects, such as neuroprotection after brain injury (Zhang et al., 2019b), improvement of myocardial ischaemia (Wang et al., 2020), anti-inflammatory and antioxidant (Jeon et al., 2020) effects. Current studies have found that the antidepressant mechanism of puerarin mainly involves the regulation of monoamine neurotransmitters, BDNF, HPA axis dysfunction and neuroinflammation. In recent years, it has been found that puerarin has strong antidepressant effects and can improve depression-like behavior in a selective nerve injury model and ovariectomy-induced mice, and has also been associated with the modulation of BDNF levels in brain tissue (Zhao et al., 2017; Tantipongpiradet et al., 2019). Moreover, puerarin can enhance the barrier function of intestinal epithelial cells and inhibit LPS leakage by increasing tight junction proteins as well as regulating microbial composition (Li et al., 2020). In addition, puerarin has been found to significantly alleviate depressive-like behaviors in chronic unpredictable mild stress (CUMS)-induced rats, and is associated with oxidative damage in the central nervous system and pro-inflammatory cytokine levels. A previous study by our group based on the antidepressant effect of Pueraria showed that both 30 mg/kg and 100 mg/kg of Pueraria were able to significantly attenuate chronic stress-induced depressive-like behaviors and improve CUMS-induced disorders of intestinal flora in mice, and showed that the higher dose group had a stronger antidepressant effect (Song et al., 2021). Although puerarin has been reported to have antidepressant effects in CUMS stress-induced rats, studies on the modulation of chronic restraint stress (CRS)-induced hepatic depression by puerarin have not yet been reported. The CRS model of depression is a hepatic depression-type animal model that is often used in current experiments of traditional Chinese medicine (TCM) research. During the modeling process, mice are restrained for a long period of time to restrict their free activities as a stress source, simulating the psychological state of tension and frustration encountered by human beings in daily life, and the long-term stress leads to emotional and emotional disorders and liver dysregulation, which is one of the commonly used modeling methods for the study of hepatic depression in traditional Chinese medicine (Han, 2021). Based on the above studies, Pueraria has been found to be effective in preventing and treating depression, but the key bacteria and potential mechanisms of its antidepressant effects have not been fully elucidated. Therefore, in this study, we proposed to establish a CRS mouse model of liver depression, and assessed the depression-like behaviors of mice from the aspect of “gut-liver-brain axis” by using five behaviors, such as lack of pleasure, behavioral depression, and delayed food intake, etc. The microbial composition of the intestinal tract of the mice was analyzed by 16S rRNA sequencing method, and the key antidepressant bacterial species were investigated. Neuroinflammation generation was inferred from changes in colonic tissues, liver function, and systemic LPS levels, and then the molecular mechanisms were elucidated in terms of inflammatory responses mediated by the TLR4/MYD88/NF-κB signaling pathway, so as to assess the effects of gerberellins on CRS-induced depressive-like behaviors in mice.

## 2 Materials and methods

### 2.1 Animals and experimental design

Puerarin was purchased from Shanghai Macklin Biochemical Technology Co., Ltd. Male ICR mice (22–26 g) were purchased from Changsha Tianqin Biotechnology Co., Ltd. [License Number: SCXK (Xiang) 2019-0013, China]. All mice were housed in an animal room with a room temperature of 25 ± 1 °C, relative humidity of 60 ± 5%, and a photoperiod of 12/12 h. Before the start of the experiment, mice were required to be housed in the animal house for one week for acclimatization, during which they were kept to drink and eat normally, and the bedding was changed regularly. After a 7-day acclimatization period, 40 mice were randomly divided into four groups of 10 mice each according to body weight and autonomic activity distance: normal + saline group (control), CRS + saline group (CRS), CRS + fluoxetine 10 mg/kg group (fluoxetine), and CRS + puerarin 100 mg/kg group (puerarin) (Song et al., 2021). All drugs were prepared with normal saline as the solvent. The control and model groups were given saline gavage once daily. Puerarin was purchased from Aladdin Biochemical Technology (Shanghai, China), production lot L2107402. Fluoxetine was purchased from Shanghai Macklin Biochemical Technology (Shanghai, China), production lot C10221539. All animal treatment procedures were approved by the Animal Care Committee of the Yichun University, Jiangxi, China.

### 2.2 Establishment of chronic restraint stress

Chronic restraint stress-induced depression mouse model established for four consecutive weeks. When the experiment started, except for the normal group, mice were placed in 50 mL centrifuge tubes restrained with 15–20 small holes to maintain air circulation, and restrained with no water and no food during the restraint period from 9:00 a.m. to 13:00 p.m. daily. The drug was administered 30 min before CRS modeling each day. The control mice were housed in five cages each without any stimulation, and the control mice were fed and watered freely without restriction every day, except for the control group, which was housed in a single cage until the end of the experiment, which lasted 28 days. Behavioral tests and biological experiments were performed after the last CRS modeling session.

### 2.3 Anesthesia description

Place the mouse in an induction chamber for anesthesia, with the isoflurane concentration set at 4–5% and the oxygen flow rate at 1–2 L/min. The mouse will lose consciousness within 2–3 min.

### 2.4 Behavioral test

Before each test, the mice were placed in the experimental environment for 1 h. After each test, the equipment needed to be sprayed and wiped with 75% alcohol.

#### 2.4.1 Sucrose preference test

Before the experiment, mice were required to acclimatize to 1% (w/v) sucrose solution for 72 h: two bottles containing 1% sucrose solution were placed in each cage, and after 24 h, the 1% sucrose solution in one of the bottles was replaced with pure water and left for 24 h. After acclimatization, mice were fasted from water and food for 24 h. For the sucrose preference test, in the experiment, mice were free to drink two bottles of water, one bottle of 1% sucrose solution 100 mL, and the other bottle was 100 mL of pure water, and the positions of the two bottles were frequently exchanged (left or right side). Twelve hours later, the remaining amount of liquid was measured, and the intake of sucrose solution and pure water of mice in 12 h was obtained, and sucrose preference (%) = sucrose solution intake/(sucrose solution intake + pure water intake) × 100%.

#### 2.4.2 Open field test

Before starting the experiment, make sure that the test chamber is clean and odour free. In order to eliminate olfactory cues, the bottom of the chamber was cleaned with 75% alcohol after each test to remove feces, urine, etc., left behind by the animals tested in the previous experiment. The bottom and four walls of the open chamber (125 × 125 × 40 cm) were black, and the bottom of the chamber was divided into 16 squares of 4 × 4 compartments. Mice were placed alone in the centre of the open box and allowed to explore freely. The number of mice crossing the grid horizontally (the number of mice crossing the grid horizontally when they entered the square with three or more paws) and the number of times they stood upright (the number of times the mice stood upright when their two front paws were in the air or climbed up the wall of the box) were recorded for a period of 5 min.

#### 2.4.3 Tail suspension test

The middle part of the tails of the experimental mice was fixed with tape downwards on a fixation rod with an equidistant iron hook. Firm pressure was applied to prevent the mice from falling during the suspension process. The mice were separated from each other by a blackboard to avoid mutual interference, and the activity of the mice was recorded by a video camera for 5 min, and the cumulative immobilisation time of the mice was recorded for the last 4 min.

#### 2.4.4 Forced swim test

The experimental equipment was a beaker 20 cm high and 14 cm in diameter. The day before the experiment, each mouse was individually placed in a beaker (14 cm in diameter and 20 cm in height) filled with 15 cm of water (24 ± 1 °C) for a 15 min pre-test of swimming, so that the mice’s hind limbs could not touch the bottom of the beaker to support their bodies. On the day of the experiment, the beaker was filled with water of the same temperature and height, and the mice were allowed to swim and filmed by a video camera for 5 min. The water was replaced after each test. The cumulative immobilisation time of the mice was recorded for the last 4 min. Mice were considered immobile when they floated on the water surface without struggling at all, or when only their hind limbs did some slight bobbing to support the head floating on the water surface. Each group of test mice was wiped with a dry towel at the end of the experiment.

#### 2.4.5 Novelty suspended feeding test

The mice were fasted for 24 h (with unlimited water intake) and were placed in a new environment (different from the environment in which the mice were housed and the other test environments) for 30 min prior to the start of the test. The 125 × 125 × 40 cm test box was lined with clean bedding about 1 cm thick, and five feed pellets were placed on a piece of filter paper (10 cm in diameter) in the middle of the test box. Mice were placed facing the wall of the box and the latency to the first ingestion within 5 min was recorded (ingestion was defined as the beginning of chewing, not simply sniffing or touching the food). The latency to ingestion for those who did not eat within 5 min was recorded as 5 min.

### 2.5 16S rRNA gene sequencing

After modeling, 3–5 pellets of fresh mouse feces (*n* = 8 per group) were taken, placed in sterile centrifuge tubes, and immediately stored at −80 °C pending microbiological assays. Total genomic DNA was extracted from mouse fecal samples by CTAB method, and the final concentration and purity of DNA were determined by NanoDrop 2000 UV–Vis spectrophotometer from Thermo Fisher Science, United States, and the DNA quality was determined by 1% agarose gel electrophoresis. And the purity and concentration of DNA were checked by 1% agarose gel electrophoresis. PCR amplification of the V4 variable region was performed using primers 515F (5′-GTGCCAGCMGCCGCGGTAA-3′) and 806R (5′-GGACTACHVGGGTWTCTAAT-3′). The library was constructed using the NEB Next^®^ Ultra DNA Library Prep Kit according to standard operating procedures, and the library was tested and quantified by Q-PCR using Agilent 5400; after the library was qualified, the library was sequenced using NovaSeq 6000. DNA extraction, PCR amplification and sequencing were entrusted to Shenzhen Microecological Science and Technology Co., Ltd. (Guangdong, China).

### 2.6 H&E staining

An appropriate amount of colon and liver tissues specimens were fixed in 4% paraformaldehyde at room temperature, dehydrated and embedded in paraffin, cut into 5-μm sections transversely with a microtome, stained with hematoxylin and eosin (H&E), and examined microscopically after passing through dehydration, transparency and sealing.

### 2.7 Enzyme-linked immunosorbent assay

The concentration of 5-HT in the PFC and the concentration of LPS in colon, serum, liver and PFC tissue were detected by enzyme-linked immunosorbent assay (ELISA) kits (Shanghai Keshun Biotechnology Co., Ltd., Shanghai, China). According to this instruction manual, all experimental procedures were performed.

### 2.8 Western blot

The prefrontal cortical tissue samples were removed from the −20 °C refrigerator, 80–100 mg of tissue was weighed and placed in a homogenization tube, 1 mL of cell lysis solution containing protease inhibitor was added to each 100 mg of sample, and under ice-water bath conditions, the homogenization tube was held in the left hand and the lower end was inserted into a vessel containing ice-water mixture, the right hand inserted the pounding rod vertically into the cannula, and the grinding was rotated up and down for dozens of times (6–8 min). The prepared homogenate was transferred to a 1.5 mL centrifuge tube with a pipette at 12,000 rpm/min and centrifuged at 4 °C for 10 min, and the supernatant was taken out and the protein concentration was determined using the Bradford method. An appropriate amount of sample was taken and mixed with 1/5 volume of 5× loading buffer of the sample and boiled for 5 min. After SDS-PAGE electrophoresis, membrane transfer, PVDF membranes were blocked in 5% skimmed milk for 1 h at room temperature, and incubated overnight at 4 °C with primary antibodies, including TLR4, MYD88, IκB-α, P-IκB-α, NF-κB (p65), p-NF-κB (p-p65) (Wanleibio. China) (1:500, 8040196, 3232494, 9221936, 9082495, 7111273, 8242169), BDNF and β-actin (Beijing 4A Biotech Co., Ltd., China) (1:500, 20200611, JJ1231). The next day, after washing the membrane with TBST, it was incubated with horseradish peroxidase (HRP)-labeled antibody: goat anti-rabbit secondary antibody (Beijing 4A Biotech Co., Ltd., China) (1:10,000, SPH102-500) at room temperature for 1 h, and then wash three times. The PVDF membrane was then placed horizontally on the chromogenic tray of AlphaImager HP gel imager, an appropriate amount of ultrasensitive ECL chemiluminescent solution mixed in the ratio of 1:1 was added dropwise, left for 1 min, and the target band chemiluminescence was detected by Alpha FluorChem gel imager and photographed and saved. Finally, the protein bands in the images were analyzed by Image J software for grayscale values.

### 2.9 Statistical analysis

All experimental data were plotted using GraphPad Prism 8.0.2 (San Diego, United States) software. Image-J software was used for grey scale analysis of WB bands. All data results were expressed as mean ± standard deviation (mean ± SD). A value of *p* < 0.05 was considered statistically significant. Data comparisons among multiple groups were performed using one-way analysis of variance with Tukey’s *post-hoc* test (ANOVA).

## 3 Results

### 3.1 Puerarin ameliorated CRS-induced depressive-like behaviors

**Figure 1:**
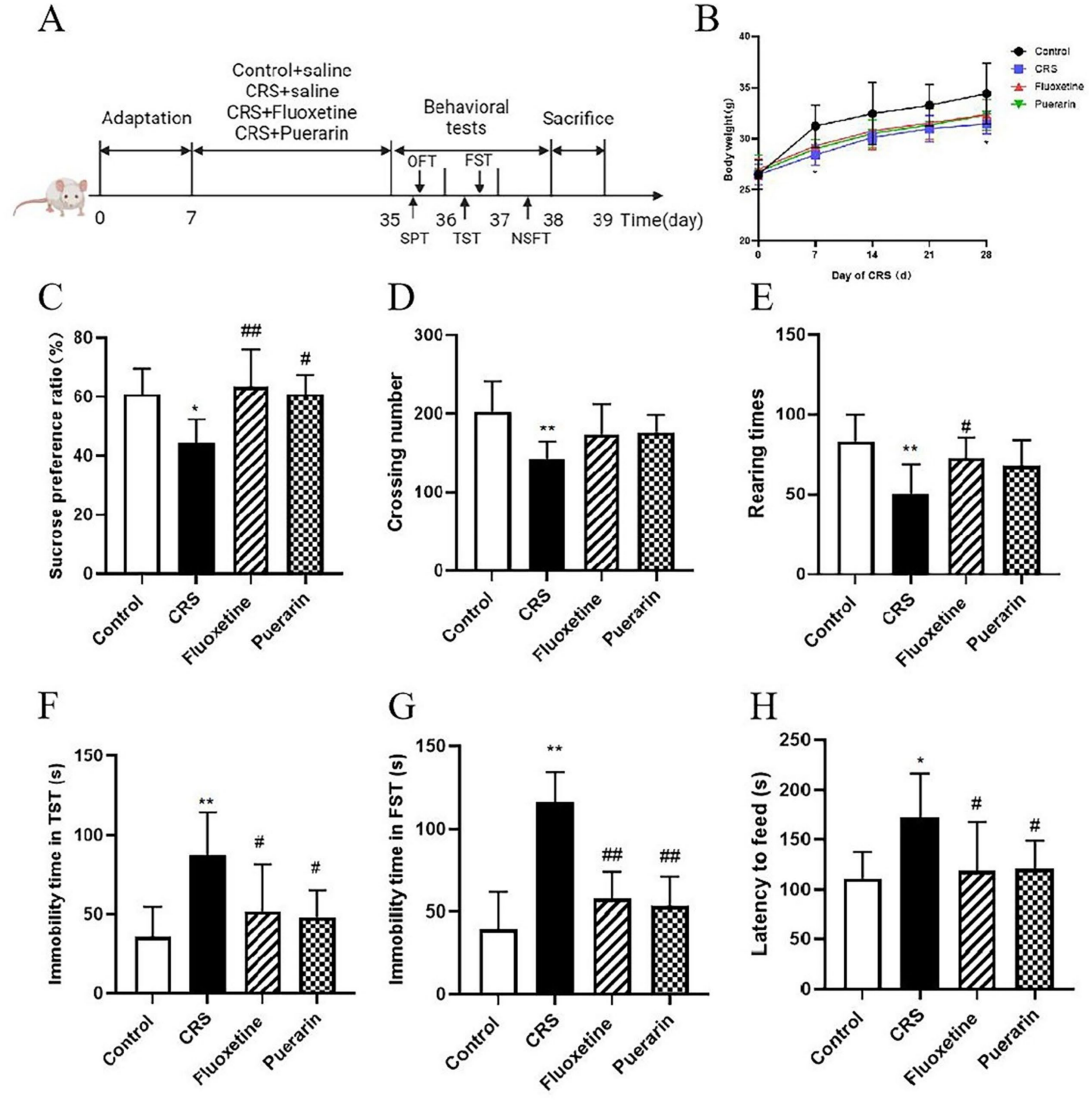
Depressive-like behavior induced by CRS and the protective effect of puerarin. **(A)** Experimental flowchart. **(B)** Body weight. **(C)** Sucrose preference ratio in SPT. **(D)** Crossing number in OFT. **(E)** Rearing times in OFT. **(F)** Immobility time in TST. **(G)** Immobility time in FST. **(H)** Latency to feed in NSFT. Data are expressed as mean ± SD. *n* = 8. ^*^p < 0.05 and ^**^p < 0.01, compared with the control group; ^#^*p* < 0.05 and ^##^*p* < 0.01, compared with the CRS group.

As illustrated in the experimental scheme (Figure 1A), behavioral tests were performed after the 28-day CRS modeling. As shown in Figure 1B, the body weight gain of mice in the model group was consistently lower than that of the control and puerarin intervention groups. The differences between the groups were not statistically significant (*p* > 0.05) except that the body weight of the model group was lower than that of the normal group on days 7 and 28 after CRS (*p* < 0.05). Pleasure deficit is one of the core symptoms of depression and can be reflected by SPT. Decreased sugar-water preference rate can reflect the loss of interest in rewarding stimuli in depressed mice, which is also a typical manifestation of pleasure deficit in mice. The results are shown in Figure 1C, SPT was performed alone, and the mice in the CRS group had a decreased rate of sugar-water preference compared with the control group (*p* < 0.05). While the fluoxetine and puerarin groups showed an increase in sugar-water preference rate compared to the CRS group (*p* < 0.01, *p* < 0.05). The results suggest that puerarin intervention can improve pleasure deficit behavior in stressed mice. The OFT are often used to measure the locomotor and exploratory behaviors of mice in novel environments. Compared with the control group, mice in the CRS group showed a significant decrease in the number of horizontal cross-grids and the number of uprights (*p* < 0.01), and an increase in the number of uprights in the fluoxetine-treated group (*p* < 0.05). Whereas, the difference in the number of horizontal cross-grids and the number of uprights of mice in the puerarin-treated group was not statistically significant (*p* > 0.05) (Figures 1D,E). In the TST (Figure 1F), the cumulative immobility time of the mice in the model group was significantly increased (*p* < 0.01) compared to the control group, and the fluoxetine and puerarin groups decreased the cumulative immobility time of the mice in the hanging tail experiment (*p* < 0.05). The results suggest that puerarin intervention can improve the depression-like behavior of stressed mice in the TST. In the FST, the cumulative immobility time was significantly increased in the CRS group compared to the control group (*p* < 0.01). Whereas, fluoxetine and puerarin treatment group significantly shortened the cumulative immobility time in the FST in depressed mice (*p* < 0.01). It suggests that fluoxetine and puerarin can significantly improve the depression-like behavior of mice in the forced swimming experiment (Figure 1G). As shown in Figure 1H, in NSFT, the CRS model group showed an increase in ingestion latency compared to the control group (*p* < 0.05). The ingestion latency was shortened in the fluoxetine and puerarin groups compared to the model group (*p* < 0.05).

### 3.2 Puerarin modulates the microbial composition of CRS mice

#### 3.2.1 Diversity analysis

**Figure 2:**
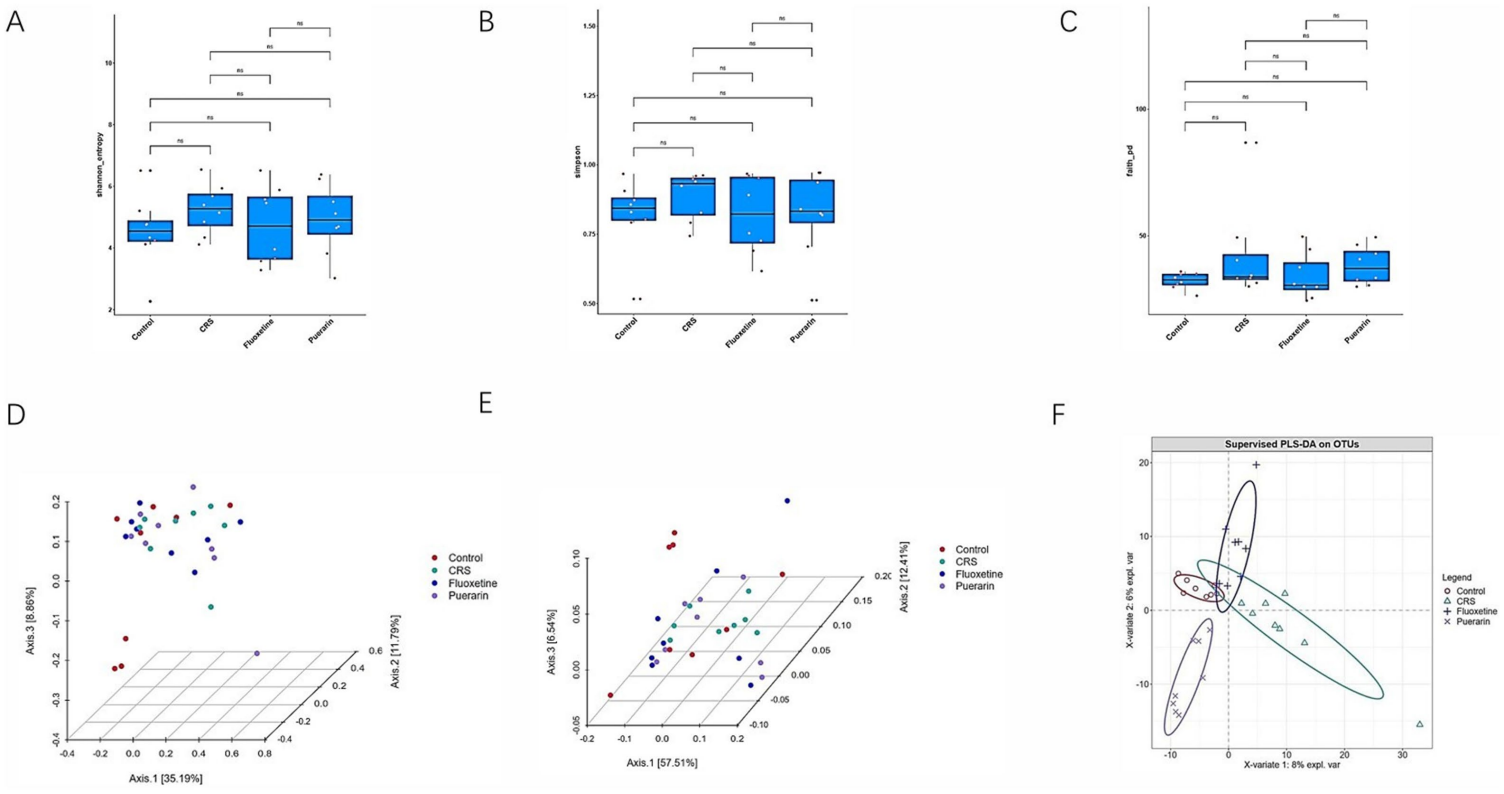
Intergroup diversity analysis of intestinal flora. **(A)** Shannon index. **(B)** Simpson index. **(C)** Faith_pd index. **(D)** Bray–Curtis distance. **(E)** Weighted UniFrac distance. **(F)** PLS-DA analysis. Ns, no significance, Wilcox_Test.

Previous studies have shown that alterations in the gut microbiota affect depressive-like behavior. Therefore, we sought to determine whether CRS-induced depressed mice exhibit changes in gut flora diversity. The abundance and diversity of the gut microbiota of four groups of mice were analzsed. As shown in Figures 2A–C, α-diversity analysis was performed by calculating Shannon, Simpson, and Faith’s phylogenetic diversity (Faith_pd) at the OTU level. The results showed that CRS-induced fecal microbial community diversity in depressed mice did not change significantly. In the β-diversity analysis, the gut microbiota of the four groups were not completely clustered together on the PCoA plots (Figures 2D,E), but the results showed no significant differences among the groups. In contrast, according to PLS-DA analysis (Figure 2F), the mouse gut microbiota clearly differentiated into four separate taxa, indicating that there may be some differences in the composition of the gut microbiota among them.

#### 3.2.2 Analysis of the species composition of the intestinal flora

**Figure 3:**
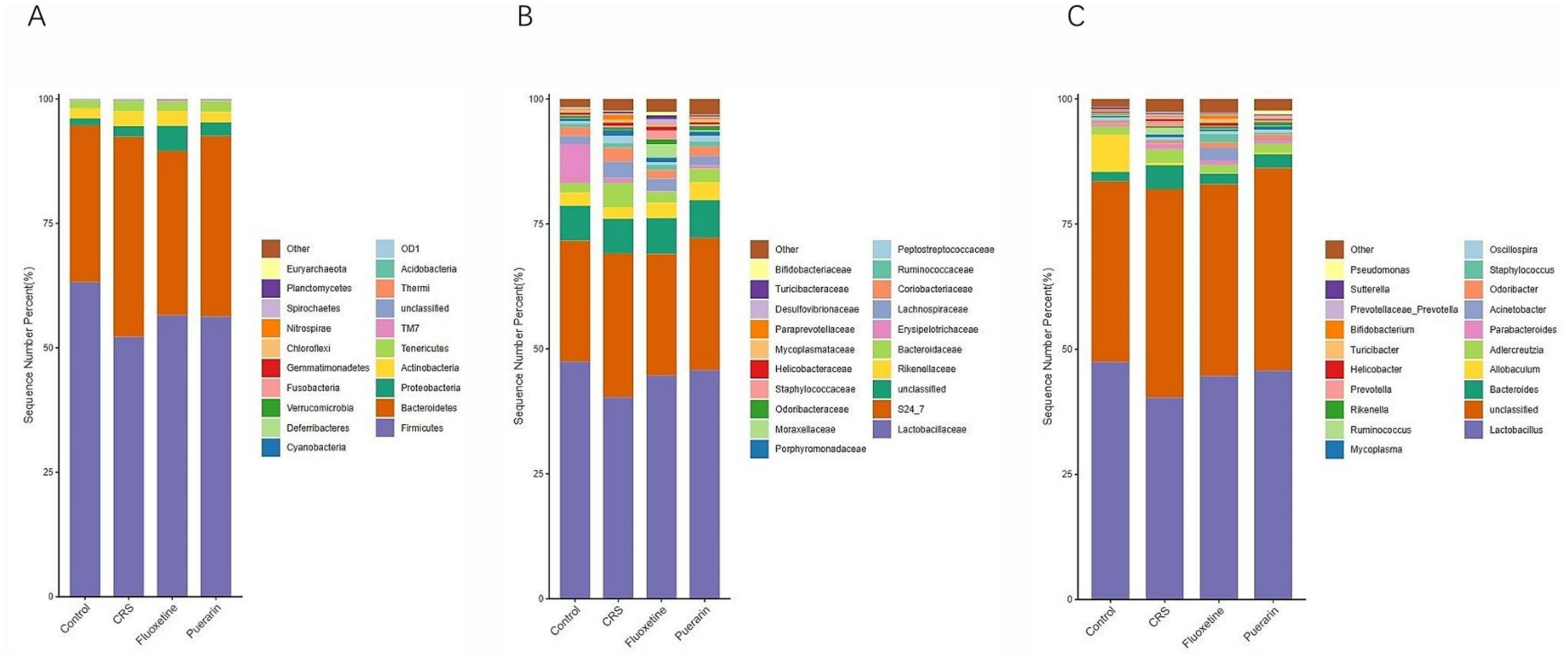
Changes in the microbial relative abundance in fecal samples from the four groups at the phylum, family, and genus levels. **(A)** Relative abundance of intestinal flora at the phylum level. **(B)** Relative abundance of intestinal flora at the family level. **(C)** Relative abundance of intestinal flora at the genus level. *Y*-axis represents relative abundance of flora.

Further analyses revealed differential bacterial taxa in all groups at the phylum, family and genus levels. At the phylum level, the abundance of Firmicutes was decreased in the model group compared to the normal control group, whereas the abundance of Bacteroidetes, Acidobacteria, Verrucomicrobia and Fusobacteria was increased; compared with the model group, the abundance of Firmicutes abundance was increased in the fluoxetine and puerarin groups compared to the model group, whereas Bacteroidetes, Acidobacteria, Verrucomicrobia and Fusobacteria abundance was decreased compared to the model group (Figure 3A).

At the family level, the abundance of Lactobacillaceae, Moraxellaceae and Rikenellaceae decreased in the model group compared to the normal control group, while the abundance of Bacteroidaceae, Lachnospiraceae, Coriobacteriaceae, and Ruminococcaceae were increased. This change could be reversed in the fluoxetine and puerarin groups compared with the model group, and in addition, the abundance of Bifidobacteriaceae was increased in the fluoxetine group compared with the model group, and the abundance of Desulfovibrionaceae was decreased in the puerarin group compared with the model group (Figure 3B).

At the genus level, the abundance of Lactobacillus and Acinetobacter was reduced in the model group compared to the normal control group, whereas the abundance of Bacteroides, Parabacteroides, Adlercreutzia, Ruminococcus and Prevotella was significantly higher. This change could be reversed in the fluoxetine and puerarin groups compared to the model group (Figure 3C), suggesting that puerarin administration ameliorated CRS-induced intestinal flora dysbiosis in depressed mice.

#### 3.2.3 Differential analysis of intestinal flora

**Figure 4:**
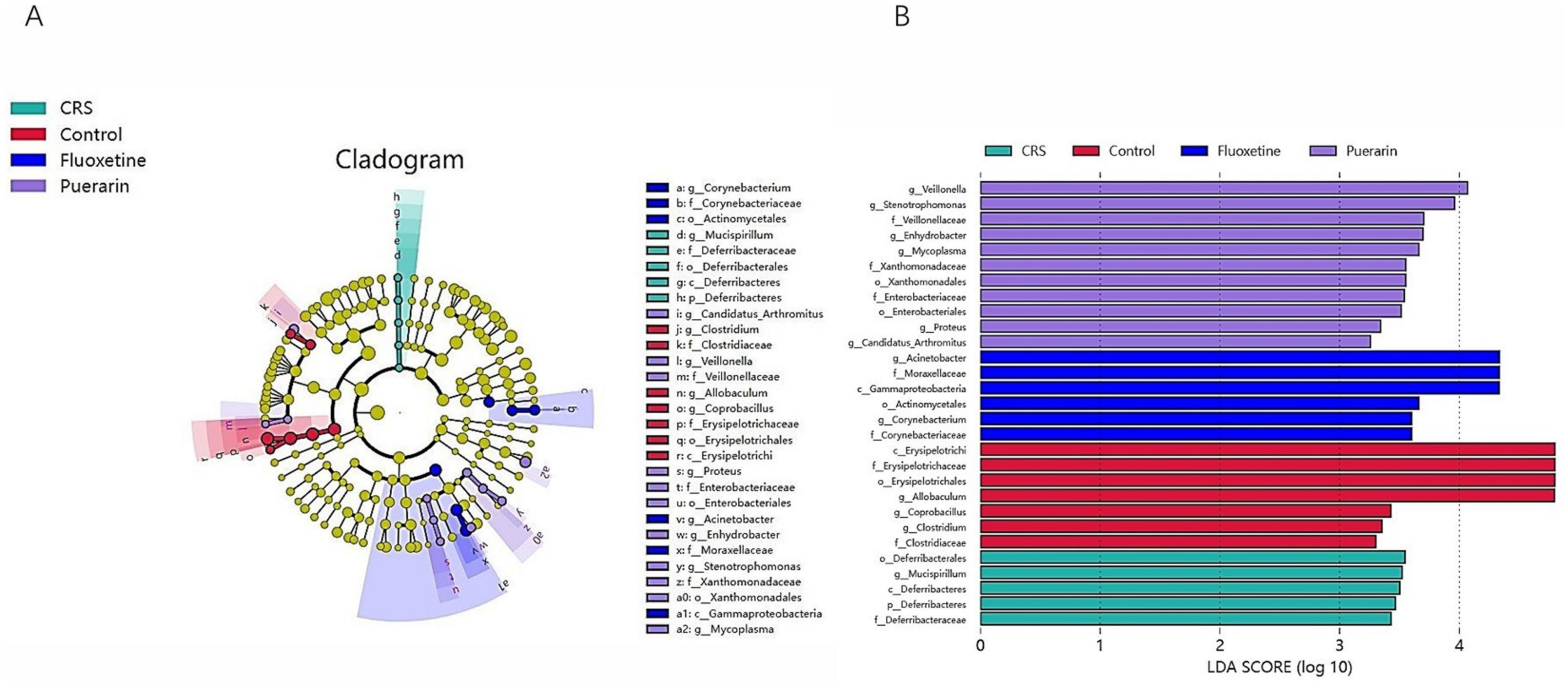
Dominant bacteria taxa in different group were identified using LEfSe. In total, 49 bacteria taxa with statistically significant and biologically consistent differences were found. **(A)** Cladogram. The size of each node represents the relative abundance of the species (p, phylum; c, class; o, order; f, family; g, genus; s, species). **(B)** Distribution histogram. The LDA score >2. The higher the LDA value, the more significant the gut microbiota in the comparison.

In order to identify dominant bacterial taxa in different groups, this study used LEfSe, a new method for identifying macro-genomic biomarkers through class comparisons, and the LEfSe evolutionary branching map showed that a total of 29 bacterial taxa with statistically significant and biologically coherent differences were identified (Figure 4A). Among them, seven bacterial taxa were significantly enriched in the control group, including c_Erysipelotrichi, f_Erysipelotrichaceae, Danitomycetes o_Erysipelotrichales, g_Allobaculum, g_Coprobacillus, g_Clostridium and f_Clostridiaceae. Five bacterial taxa were significantly enriched in the model group, including o_Deferribacterales, g_Mucispirillum, c_Deferribacteres, p_Deferribacteres, and f_Deferribacteraceae, and the LDA threshold was relatively low. Bacterial enrichment and abundance increased significantly after fluoxetine and puerarin interventions, with six bacterial taxa significantly enriched in the fluoxetine group, including g_Acinetobacter, f_Moraxellaceae, c_Gammaproteobacteria, o_Actinomycetales, g_Corynebacteriu and f_Corynebacteriaceae. Eleven bacterial taxa were significantly enriched in the Graminome, mainly including g_Veillonella, g_Stenotrophomonas, f_Veillonellaceae, g_Enhydrobacter, g_Mycoplasma, f_Xanthomonadaceae, o_ Xanthomonadales, f_Enterobacteriaceae, o_Enterobacteriales, g_Proteus, g_Candidatus_Arthromitus (Figures 4A,B). LEfSe bar analysis further showed that gut flora enrichment was reduced in mice after external stimuli and that altering gut flora enrichment after puerarin intervention alleviated depressive symptoms.

### 3.3 Puerarin attenuates CRS-induced colonic tissue damage in mice

**Figure 5:**
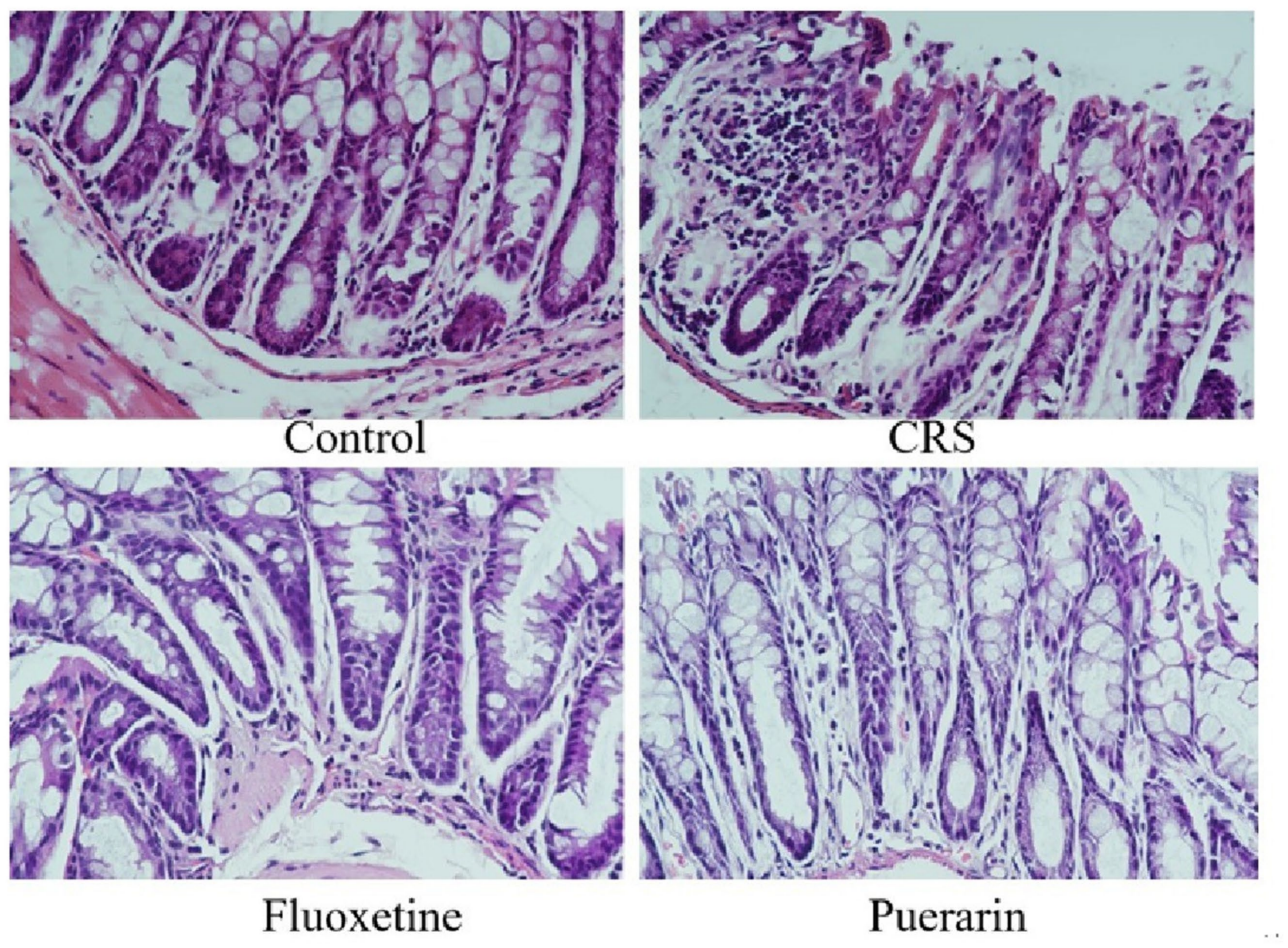
Effects of puerarin on histopathological changes in the colon of CRS-induced mice (H&E staining, 400×).

Histopathology of the colon showed (Figure 5) that the structures of the mucosal layer, submucosal layer and muscularis layer of the control colon tissue were clear and intact. The intestinal villi were regularly arranged, and the intestinal glands consisted of multiple cells that were tightly arranged. The muscle fibres of the muscular layer were regularly arranged and no obvious abnormalities were seen. The intestinal epithelial structure in the mucosal layer of the colon of mice after CRS modeling was disrupted, the epithelial cells were loosely arranged, and a small number of cells had consolidated deeply stained, fragmented or dissolved nuclei, with obvious inflammation. The overall normal colonic tissues in the fluoxetine and puerarin groups, with intestinal epithelial structure intact, tightly arranged and morphologically normal epithelial cells, and a large number of cup cells in the intestinal lamina propria, partially inhibited CRS stress-induced colonic tissue injury.

### 3.4 Puerarin reduces LPS levels in colon, serum, liver, and PFC

**Figure 6:**
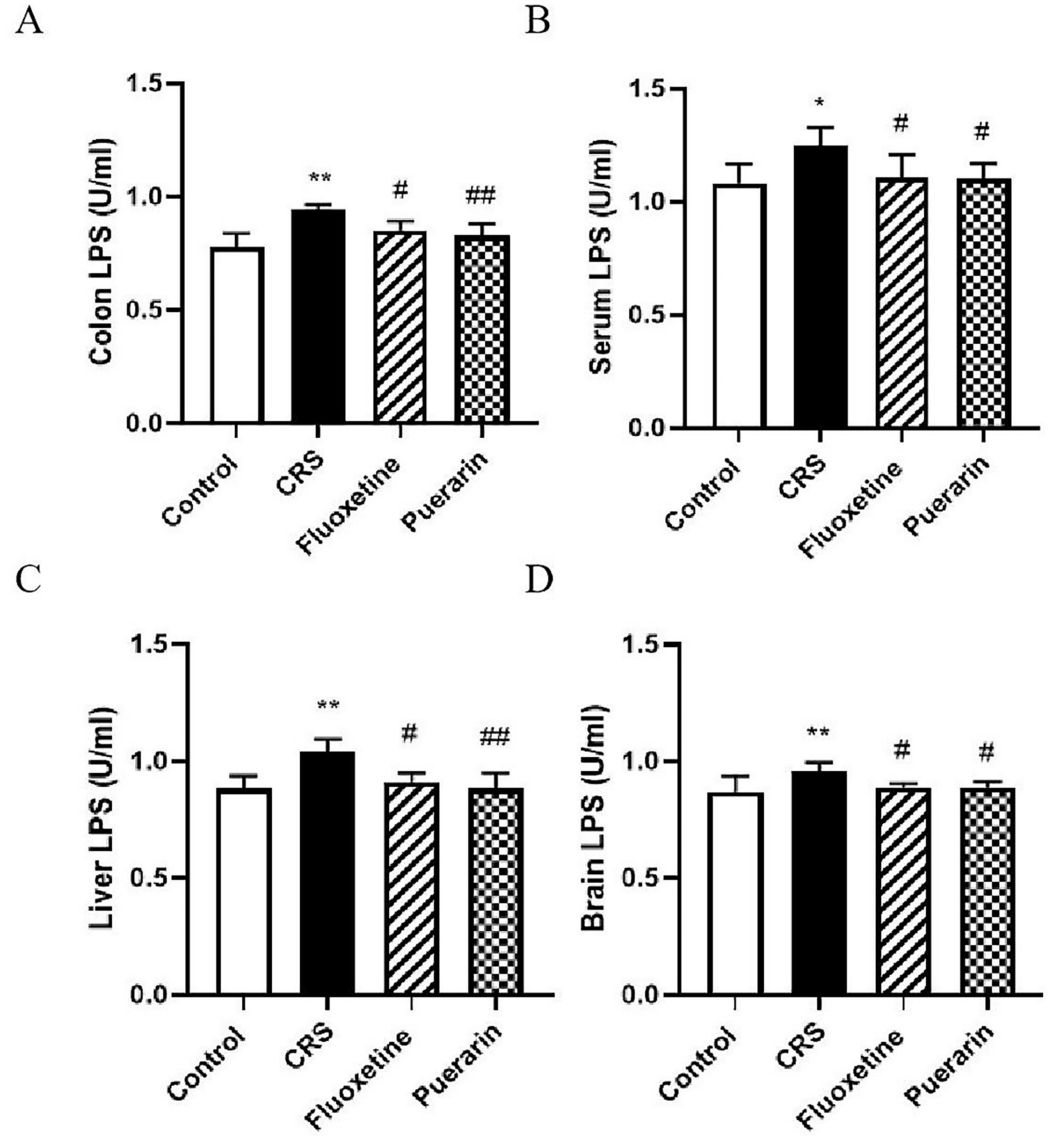
Effects of puerarin on LPS levels in colon, serum, liver, and brain in CRS-induced mice. **(A)** Colon was analyzed for LPS. **(B)** Serum was analyzed for LPS. **(C)** Liver was analyzed for LPS. **(D)** Brain was analyzed for LPS. Data are expressed as mean ± SD. *n* = 8. ^*^p < 0.05 and ^**^p < 0.01, compared with the control group; ^#^*p* < 0.05 and ^##^*p* < 0.01, compared with the CRS group.

The intestinal epithelium is a barrier that prevents the translocation of bacterial-derived factors. Chronic restraint stress disrupts the intestinal flora and disrupts the intestinal barrier function in mice, which results in the translocation of the bacterial-derived factor LPS, leading to elevated levels of LPS in the blood. The experimental results showed that LPS levels were elevated in the colon, serum, liver and prefrontal cortex of CRS stress-induced mice compared with normal controls (Figures 6A–D). It is suggested that LPS may be traveling through the hepatic and intestinal circulation, into the body circulation, and that some LPS crosses the BBB into the centre, leading to the development of central chronic inflammation. Whereas, after administration of fluoxetine and puerarin, LPS levels were reduced in colon (*p* < 0.05, *p* < 0.01), serum (*p* < 0.05), liver (*p* < 0.05, *p* < 0.01), and prefrontal cortex (*p* < 0.05) compared to the model group. It suggests that puerarin reduces CRS-induced systemic LPS levels and thus has an intervention effect on the body inflammation generated by high levels of LPS.

### 3.5 Puerarin attenuates CRS-induced liver injury in mice

**Figure 7:**
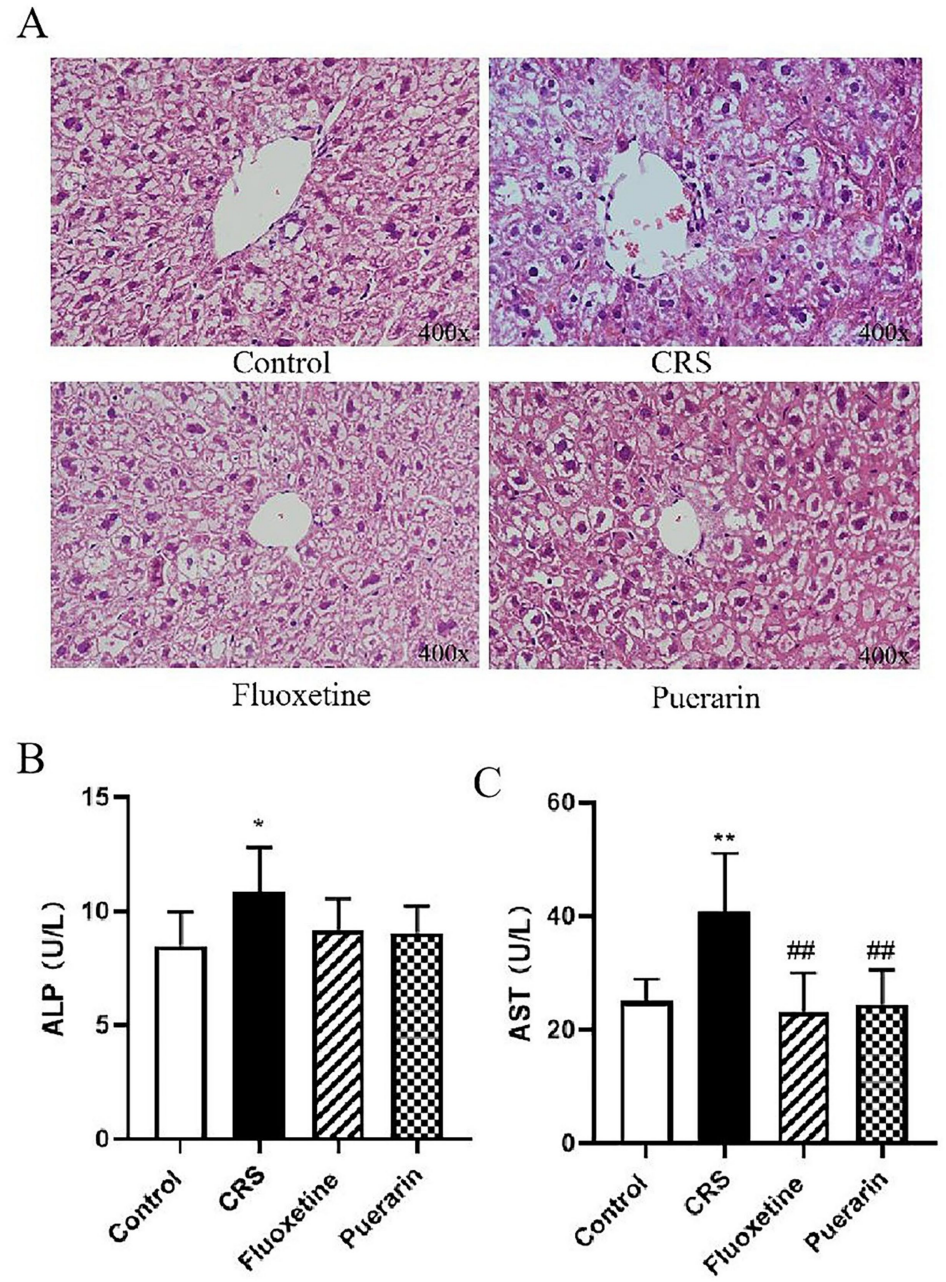
Effect of puerarin on liver function indexes of mice induced by CRS. **(A)** Liver histopathology was performed by H&E staining (400×). **(B)** Serum was analyzed for ALP. **(C)** Serum was analyzed for AST. Data are expressed as mean ± SD. *n* = 8. ^*^p < 0.05 and ^**^p < 0.01, compared with the control group; ^##^*p* < 0.01, compared with the CRS group.

LPS stress has been shown to increase the expression of TLR4 and downstream signaling components in various tissues. High levels of LPS in the organism may cause severe inflammation and cell death, and the liver, which is the first target of portal LPS, is the main organ involved. Histopathological analysis of the liver showed that the liver tissue of the normal group had well-defined and well-arranged hepatic lobules, hepatocytes were arranged radially with a central vein, hepatocytes had a full morphology, and no obvious inflammatory changes were observed. The liver tissue of mice in the model group had a large number of hepatocytes with vacuolar degeneration, a larger number of hepatocytes with sparse or vacuolated cytoplasm, and local small foci of hepatocyte necrosis with fragmented nuclei and enhanced cytoplasmic eosinophilicity with haemorrhage and a small amount of inflammatory cell infiltration. Liver tissue in the fluoxetine and puerarin groups was overall normal and partially suppressed the histopathological damage of the liver (Figure 7A). Compared with the control group, the serum levels of ALP and AST were elevated in the model group mice (*p* < 0.05, *p* < 0.01). It was suggested that the liver function of the mice in the model group was abnormal and there was some liver damage. Compared with the model group, the administration of fluoxetine and puerarin decreased the AST content (*p* < 0.01), while the ALP content also had a tendency to regress (Figures 7B,C), indicating that puerarin could improve some of the hepatic function indexes in depressed mice.

### 3.6 Puerarin regulates TLR4/MYD88/NF-κB signaling pathway in the PFC of mice

**Figure 8:**
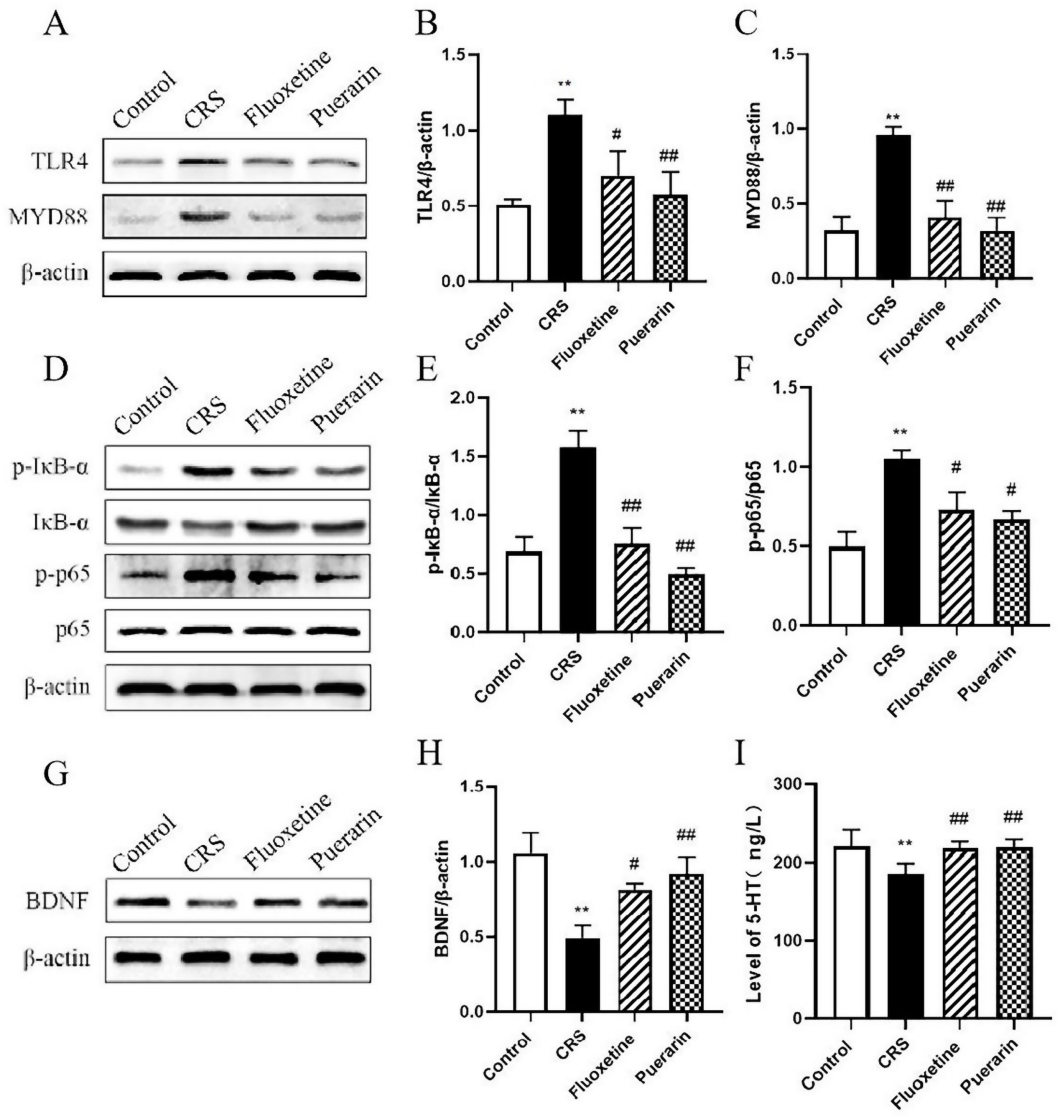
Effects of puerarin on the expression of TLR4/MYD88/NF-κB signaling pathway, BDNF, and 5-HT in PFC of CRS-induced mice. **(A)** Representative Western blot brands of TLR4 and MYD88 in PFC. **(B)** The contents of TLR4 expression in PFC. **(C)** The contents of MYD88 expression in PFC. **(D)** Representative Western blot brands of p-IκB-α, IκB-α, p-p65, and p65 in PFC. **(E)** The ratio of p-IκB-α/IκB-α in PFC. **(F)** The ratio of p-p65/p65 in PFC. **(G)** Representative Western blot brand of BDNF protein in PFC. **(H)** The contents of BDNF expression in PFC. **(I)** The contents of 5-HT expression in PFC. Data are expressed as mean ± SD. *n* = 8. ^*^p < 0.05 and ^**^p < 0.01, compared with the control group; ^#^*p* < 0.05 and ^##^*p* < 0.01, compared with the CRS group.

LPS can induce inflammation through TLR4/MYD88/NF-κB, and Western blot results showed that the expression of TLR4 and MyD88 proteins in the prefrontal cortex of mice induced by CRS stress was significantly higher than that in the non-stressed control group (*p* < 0.01). Both TLR4 (*p* < 0.05, *p* < 0.01) and MYD88 protein (*p* < 0.01) expression was reversed in the prefrontal cortex of mice after fluoxetine and puerarin administration compared with the model group (Figures 8A–C). Next, we examined the phosphorylated expression of IκB-α and p65 proteins in the prefrontal cortex of mice using WB as well, and the expression levels of p-IκB-α/IκB-α and p-p65/p65 were significantly elevated in the prefrontal cortex of mice in the model group compared to the control group (*p* < 0.01). In contrast, fluoxetine and puerarin administration decreased IκB-α (*p* < 0.01) and p65 (*p* < 0.05) phosphorylation expression in the prefrontal cortex of mice (Figures 8D–F). Taken together, puerarin may attenuate neuroinflammatory damage and ultimately improve depressive symptoms in CRS mice by inhibiting the overactivation of the TLR4/MYD88/NF-κB inflammatory signaling pathway by LPS in the prefrontal cortex of depressed mice.

### 3.7 Puerarin modulates BDNF and 5-HT levels in the PFC of mice

Figures 8G,H shows that CRS-induced BDNF expression in the prefrontal cortex of mice was significantly reduced compared to the control group (*p* < 0.01), whereas fluoxetine and puerarin treatments increased BDNF expression in the prefrontal cortex compared to the model group (*p* < 0.05, *p* < 0.01). It suggests that puerarin can ameliorate the CRS stress-induced decrease in BDNF expression. The relative levels of monoamine neurotransmitters have been used as an important indicator for assessing depression. Therefore, the levels of the monoamine neurotransmitter 5-HT associated with the positive control drug fluoxetine were experimentally examined in the prefrontal cortex of mice. The results showed that 5-HT levels in the prefrontal cortex of mice in the model group were significantly reduced (*p* < 0.01) compared to the control mice, and the administration of fluoxetine and puerarin significantly increased 5-HT levels in the prefrontal cortex of the mice compared to the model group (*p* < 0.01) (Figure 8I). It suggests that puerarin can ameliorate the CRS-induced decrease in 5-HT levels in the prefrontal cortex of mice, thereby exerting an antidepressant effect.

## 4 Discussion

Depression is one of the most notable public health issues facing the world, and evidence suggests that depression may be linked to the gut microbiome (Cheung et al., 2019). The balance of the human gut flora can be disturbed by stress, which in turn can lead to a range of subsequent mental health problems such as anxiety and depression (Ma et al., 2021). The gut microbiome consists of a large bacterial and viral community that influences the development of the central nervous system having an impact on host health and disease (Yang et al., 2020). The current animal models of liver depression-related depression used in research experiments for TCM are basically CRS animal models. CRS is a valid and reliable model of depression and is now widely used in animal models of depression (Wang et al., 2022). Puerarin has a wide range of pharmacological effects, such as anti-inflammatory and antioxidant effects, and in recent years, it has been found that puerarin has strong antidepressant effects (Qiu et al., 2017), but there are few studies based on the CRS-induced hepatic depression model, and the antidepressant effects of its “gut-liver-brain axis”-related mechanisms have not yet been fully elucidated. Therefore, in the present study, we established a CRS mouse model of liver depression, and evaluated the depression-like behavior of mice from the aspect of “gut-liver-brain axis,” firstly, through various behavioral tests, and then demonstrated whether puerarin could regulate the dysfunction of intestinal barriers and dysregulation of the intestinal microbiota. The potential mechanism of Pueraria Mirifica in alleviating depression will be explored by inferring the neuroinflammation from the changes in colonic tissues, liver function and systemic LPS levels. Our results showed that puerarin administration ameliorated CRS-induced gut microbiota dysbiosis, improved damaged colon and liver tissues in depressed mice, and suppressed neuroinflammatory damage mediated by the TLR4/MYD88/NF-κB signaling pathway, alleviating depressive symptoms. And puerarin treatment effectively attenuated the depressive-like behaviors measured in SPT, TST, FST and NSFT in CRS-induced depressed mice.

“Low food intake and dullness” is one of the clinical manifestations of liver depression. It has been shown that the body weight of mice in the CRS model of liver depression is lower than that of mice in the normal control group (Jiang et al., 2022). From the clinical data, depressed patients tend to show loss of appetite leading to weight loss. In contrast, a small number of depressed patients may develop a strong appetite and experience binge eating, with a substantial short-term increase in both food intake and body weight, but may later manifest anorexia. Some studies have shown that depression is more associated with overweight people than normal weight people, which may be caused by the vicious cycle of depression and obesity in overweight people (Coryell et al., 2016). The results of body weight analysis of the mice in this experiment showed that CRS mice had reduced body weight on day 7 and day 28 compared to the control group. The decrease in body weight of CRS mice on day 7 compared to the normal group may be due to the fact that the mice were just exposed to the stressor and the organism could not cope with it in time. After receiving the stimulus for a period of time, it increased the tolerance and adaptability of the mice. Therefore, there was no significant difference in the body weight of the mice in each group on days 14 and 21, while the body weight of the CRS mice decreased again on day 28. This may be due to the fact that the mice had already been subjected to restraint stress for 4 weeks resulting in some physical and mental damage to the mice. Chinese medicine theory that “the liver is wood, the main excretion, like the organization, evil depression.” Therefore, if the liver is normal, the qi and blood will be harmonised, the mood will be relaxed, and the negative emotions can be regulated in time. On the contrary, if the liver excretory dysfunction, the qi is not smooth, appear depressed, depression and other symptoms. Therefore, mood is an important indicator to reflect whether “liver depression” or not, and persistent low mood and depression is one of the main manifestations of “liver depression” (Din, 2020). Pleasure deprivation is one of the core symptoms of depression, which can be reflected by SPT (Qiao et al., 2020). The results of SPT showed that the elevated sugar-water preference of puerarin-treated mice ameliorated CRS-induced pleasure deprivation in depressed mice compared with the model group. Meanwhile, puerarin treatment also shortened the cumulative immobility time in TST and FST as well as the ingestion latency in NSFT in CRS mice, suggesting that puerarin could improve the depressive-like behavior in depressed mice. It suggests that 4-week CRS-induced depressive hepatic depression mice have pleasure deficit and despair manifestations, and that puerarin significantly improves such behaviors and manifestations. In contrast, OFT results indicated that puerarin intervention did not significantly improve the number of horizontal cross frames and the number of uprights in depressed mice. No differences in total distance, speed of travel and centre-area distance between groups in OFT after administration of puerarin to depressed mice were also found in a recent study (Zhang et al., 2022). These results suggest that puerarin may have little effect on locomotor and exploratory behaviors in CRS-induced depressed mice in a novel environment. However, Song et al. (2022) experimented that puerarin significantly increased the number of crossing frames and the number of uprights in OFT in CUMS-induced depressed mice, suggesting that puerarin may increase the autonomous activities and exploratory behaviors of depressed mice in order to alleviate CUMS-induced depressive state.

Previous studies have reported that the diversity and stability of the microbiota are important indicators of organismal health. Gut microecological dysbiosis stimulates increased intestinal permeability, which leads to leakage of circulating microbial products, which in turn induces systemic inflammation affecting host health (Nagpal et al., 2018). Alterations in the gut microbiota are closely related to the pathogenesis of depression. The abundance and diversity of the gut microbiota is reduced by psychoinduced stress and depressive behaviors to which rodents are subjected (Winter et al., 2018). In order to identify the key bacteria and potential mechanisms of gerberellin antidepressant, the diversity and composition of the gut microbiota of CRS-induced depressed mice with and without gerberellin intervention were experimentally investigated. The results showed that chronic stress not only affects brain and immune system functions, but also disrupts the gut microbiota. Further analysis revealed that the relative abundance of Firmicutes was reduced in the model group compared to the normal control group, while the relative abundance of Bacteroidetes, Bacteroidaceae, Bacteroides and Parabacteroides was increased, and this change could be reversed in the fluoxetine group and the puerarin group compared to the model group. An increase in Firmicutes and a decrease in Bacteroidetes was observed in inflammatory vesicles NLRP3-deficient mice and NLRP3-deficient mice improved stress-induced depression-like behavior (Zhang et al., 2019a). Colon inflammation was reduced in GF mice when colonised with a microbiota enriched with *B. thicketi* (derived from healthy human donors) (Natividad et al., 2015). Kuhn et al. (2018) found that Bacteroides mimeticus order bacteria promoted IL-6 production by colonic intraepithelial IELs in a MyD88-dependent manner, leading to inflammation. Another study showed that oral treatment with *Bacteroides fragilis* corrected intestinal permeability, altered microbial composition and ameliorated behavioral deficits such as communication abnormalities, stereotyping and anxiety-like behaviors associated with Autism spectrum disorder (ASD) in mice (Hsiao et al., 2013).

The experimental results showed that the relative abundance of Lactobacillaceae and Lactobacillus decreased in the model group of mice compared with the normal control group, while the relative abundance of Ruminococcaceae, Ruminococcus and Prevotella increased. This change was reversed in the fluoxetine and puerarin groups compared to the model group. In addition, the relative abundance of Bifidobacteriaceae increased in the fluoxetine group compared to the model group, and the relative abundance of Desulfovibrionaceae decreased in the puerarin group compared to the model group. Lactobacilli belong to the Lactobacillaceae, which colonise various parts of the body such as the oral cavity and the gastrointestinal tract, and studies have shown that an increase in Lactobacillaceae correlates with the anti-inflammatory environment of the body and has the ability to inhibit inflammatory cytokines and thus protects the body from inflammatory attacks (Ojo et al., 2019) and the mechanism by which bifidobacteria inhibit the inflammatory response is associated with NF-κB activation induced by blocking LPS (Riedel et al., 2006). Bifidobacteria and Lactobacillus counts tend to be lower in fecal samples from patients with major depressive disorder (MDD) compared to healthy controls (Aizawa et al., 2016). Previous studies have shown that probiotics *Lactobacillus reuteri* NK33 and *Bifidobacterium adolescentis* NK98, isolated from healthy human feces, effectively inhibited LPS-induced NF-κB activation while increasing hippocampal BDNF expression, synergistically alleviating anxiety- and depression-like behaviors in mice (Jang et al., 2019). These findings are consistent with the idea that NF-κB is increased in response to stress and is responsible for stress-induced depression-like behavior and stress-damaged neurogenesis. Members of the control group’s dominant bacterial taxon g_Allobaculum include mucin degraders, whose relative abundance is now known to be inversely correlated with diet-induced markers of inflammation, including leptin and IL-22 (Ravussin et al., 2012; Zenewicz et al., 2013). In addition, Ruminococcus is a pathogenic bacterium, and studies have reported that antidepressants reduce the abundance of Ruminococcus in the gut of mice (Lukić et al., 2019). And Ruminococcus abundance was also increased in the gut flora of psychiatric patients (Zhuang et al., 2018). As Gram-negative bacteria, most members of Desulfovibrio are LPS producers (Zhang Q. et al., 2018). Gut microecological dysregulation results in progressive leakage from a compromised intestinal barrier, allowing LPS to enter the enterohepatic circulation, and low concentrations of LPS in the bloodstream may cause systemic and targeted inflammation by activating TLR4 signaling in various cells (Chang et al., 2015). Another study found that increased Prevotella spp. was also observed in the intestinal flora of patients with MDD (Kelly et al., 2016). These results suggest that puerarin treatment moderately improves intestinal microecological disturbances in depressed mice. Reducing harmful bacterial abundance and increasing beneficial bacterial abundance to a certain extent improved depression-like symptoms in CRS mice.

A recent study reported changes in the liver enzymes AST and ALT in patients with MDD (Liu et al., 2021). Elevated AST, ALT and ALP in plasma are typical manifestations of liver injury. Therefore, these factors are often utilised in experimental studies to assess hepatic dysfunction and the hepatoprotective activity of drugs (Sharawy et al., 2021). In this study, HE staining of the liver showed that puerarin treatment attenuated CRS-induced histopathological changes in the liver and reduced CRS-induced elevation of the liver enzyme AST, as well as a tendency to regress ALP levels in the serum of depressed mice after puerarin treatment. Previous studies have shown that serum levels of AST and ALT were significantly higher in depressed rats compared to controls (Jia et al., 2017). In addition, ALP can alleviate LPS-induced toxicity by removing the terminal phosphate groups in the lipid A structure of LPS, which reduces liver injury and pro-inflammatory cytokine expression (Wu et al., 2021). LPS also induces tissue damage in the liver of mice, as well as increasing serum levels of AST and ALT in the mice (Li et al., 2021). These data suggest that depression is closely related to liver function and that puerarin has a protective effect against CRS-induced liver injury, which may be related to the reduction of LPS levels *in vivo*.

Alterations in gut microbiological composition and changes in liver function can lead to inflammatory brain dysfunction (Liu et al., 2020). In contrast, in the pathophysiology of depression, activation of the inflammation-associated receptor TLR4 promotes the up-regulation of its own expression, and TLR4 mRNA and protein have been found to be elevated in both the peripheral and central nervous system in patients with MDD (Hung et al., 2014). LPS acts as an agonist at the TLR4 receptor, activating the TLR4/MYD88/NF-κB signaling pathway and increasing the expression of pro-inflammatory cytokines such as TNF-α, IL-1β and IL-6. Chronic stress alters BBB integrity and increases BBB permeability through loss of the tight junction protein claudin-5. Pro-inflammatory cytokines such as peripheral IL-6 and TNF-α are more likely to cross the BBB to trigger inflammation, and blockade of pro-inflammatory cytokines such as IL-6 or TNF-α decreases stress-induced BBB opening (Menard et al., 2017; Cheng et al., 2018). The anti-inflammatory effects of puerarin in peripheral inflammatory diseases have been extensively studied and may be related to the inhibition of TLR4 and its downstream signaling (Gao et al., 2021). Current research suggests that neuroinflammation plays a crucial role in the pathogenesis of depression (Yue et al., 2017). In this study, it was found that long-term application of puerarin exhibited antidepressant effects by ameliorating neuroinflammation in depressed mice in a CRS model, and the underlying mechanism may be related to the inhibition of the TLR4/MYD88/NF-κB pathway via inhibition of the TLR4/MYD88/NF-κB pathway. TLR4 is predominantly expressed in microglia in the CNS, where it induces pro-inflammatory responses in microglia to many stimuli. If the TLR4 pathway is incorrectly activated or the signal is amplified uncontrollably, the cytokine response may adversely affect the nervous system (Le et al., 2019). In contrast, TLR4-deficient mice may attenuate LPS-induced neuroinflammation and apoptosis (Chen et al., 2021). The results showed that LPS content was increased in colon, serum, liver and prefrontal cortex of depressed mice induced by CRS. However, puerarin treatment decreased LPS content and inhibited the expression of TLR4 and MYD88 proteins, as well as the phosphorylated expression of IκB-α and p65 proteins. These data suggest that puerarin may attenuate neuroinflammation by inhibiting the TLR4/MYD88/NF-κB signaling pathway, which in turn ameliorates CRS stress-induced depression-like symptoms in mice.

In addition, reduced diversity of gut flora, reduced probiotics and dysbiosis of gut flora may lead to reduced 5-HT levels (Fu et al., 2021). Abnormalities in central 5-HT neurotransmission are closely related to the pathogenesis of depression (Shen et al., 2019). In the present study, we showed that 5-HT levels were significantly reduced in the prefrontal cortex of CRS mice after puerarin intervention, suggesting that the antidepressant effect of puerarin may be mediated through central monoamine neurotransmission. In addition, a large body of evidence suggests that BDNF, the most abundant neurotrophic factor in the body, has been shown to regulate various brain functions and is one of the key factors in the pathogenesis of depressive disorders (Xiao et al., 2020). In animal studies, stress and depression reduced the expression and function of BDNF, while antidepressants promoted the expression of BDNF, which can promote neuronal differentiation and survival, as well as maintain synaptic plasticity, through the activation of tyrosine kinase B (Sun et al., 2020). Human studies have reported that reduced expression of BDNF protein was found in the brains of depressed suicidal patients (Misztak et al., 2020). The present study showed that puerarin intervention improved the level of BDNF in the prefrontal cortex compared to the model group. It suggests that the antidepressant effect of puerarin may be related to the upregulation of BDNF expression in the brain. In addition, increasing the levels of 5-HT and BDNF alleviated depression by a mechanism that may be related to gut microbes (Zhuang et al., 2022). Overall, these results suggest that puerarin can alleviate depression by modulating the levels of 5-HT and BDNF. This experiment has certain limitations. For instance, while the data indicate a strong correlation between puerarin treatment, changes in gut microbiota, and behavioral improvements, it cannot establish a causal relationship. On the other hand, the control group mice were housed socially, whereas the CRS (chronic restraint stress) mice were individually caged. This factor may also have influenced the experimental results to some extent.

## 5 Conclusion

The above results indicate that puerarin is effective in alleviating CRS-induced depression-like behaviors measured in SPT, TST, FST and NSFT in mice. Compared with the CRS model group, puerarin increased the rate of sugar-water preference in SPT and shortened the cumulative immobilisation time in TST and FST as well as the delayed period of ingestion in NSFT in depressed mice. In addition, puerarin administration ameliorated CRS-induced intestinal dysbiosis in mice by elevating the abundance of beneficial bacteria such as Lactobacillaceae and Lactobacillus, and decreasing the relative abundance of harmful bacteria such as Ruminococcaceae, Ruminococcus, Desulfovibrionaceae and Prevotella. Relative abundance. In addition, puerarin upregulated the expression of 5-HT and BDNF in the prefrontal cortex, reduced the levels of LPS, AST and ALP in depressed mice, improved the damaged colon and liver tissues in depressed mice, and inhibited the neuroinflammatory damage mediated by the TLR4/MYD88/NF-κB signaling pathway. Taken together, our results suggest that the positive preventive effect of puerarin in CRS-depressed mice is partly attributable to the fact that puerarin ameliorates CRS-induced depressive symptoms and is closely related to the “gut-liver-brain axis” mechanism. However, this study still has some limitations, such as reliance on mouse animal models and a lack of human validation. These aspects will be addressed and improved in subsequent experiments.

## Data Availability

Original datasets are available in a publicly accessible repository: https://www.jianguoyun.com/p/DQPDmY8QqOuADhiM0ZsGIAA.

## Glossary

CRS: Chronic restraint stress
SPT: Sucrose preference test
OFT: Open field test
TST: Tail suspension test
FST: Forced swim test
NSFT: Novelty suspended feeding test
5-HT: 5-Hydroxytryptamine
BDNF: Brain-derived neurotrophic factor
LPS: Lipopolysaccharide
ALP: Alkaline phosphatase
AST: Aspartate aminotransferase
TLR4: Toll-like receptor 4
IκB-α: Inhibitor α of NF-κB
NF-κB: Nuclear factor-kappa B
BBB: Blood-brain barrier
PCoA: Principal coordinate analyses
PLS-DA: Partial least squares discriminant analysis
LDA: Linear discriminant analysis
LEfSe: Linear discriminant analysis effect size
ANOVA: One-way analysis of variance
ELISA: Enzyme-linked immunosorbent assay
